# The Average UK Adult Reading Age Is Not 9 Years: A Response to a Widely Cited Health Literacy Claim

**DOI:** 10.1111/hex.70909

**Published:** 2026-11-10

**Authors:** George Altman, Sara Salman, Fiona Watson, Mike Oliver, Gill Rowlands

**Affiliations:** ^1^ Halton Borough Council Cheshire UK; ^2^ North Cheshire and Mersey NHS Foundation Trust Warrington UK; ^3^ Health Literacy UK CIC UK; ^4^ Health Psychology Matters Ltd UK; ^5^ Public Health Sciences Institute Newcastle University Newcastle Upon Tyne UK

## Patient and Public Involvement

As this letter to the editor did not include any primary research, direct patient and public involvement was not sought.

Dear Professor Chew‐Graham,

We read with interest the paper by Haider and Wallace examining the readability of fitness to practise documents written by UK health and social care regulators [1]. This study provides new data addressing an important and neglected determinant of regulatory accessibility.

We would, however, like to comment on the claim underpinning the results, that *the average reading age of adults in the United Kingdom is typical of a child in UK education at 9 years old*. While similar statements have been widely repeated in the media and grey literature, this statement is not supported by the three citations given. We believe that the claim has become an unhelpful urban myth.

We could not identify any national surveys which directly measure reading age. The most recent English population‐level survey that measures literacy and compares to an age equivalent is the 2011 Skills for Life Survey [2]. However, the survey uses a test that includes both reading and writing. This survey uses five categories based on the national qualifications framework (NQF) as shown in Table 1. There are three entry levels: Entry Level 1 (5–7 years old), Entry Level 2 (7–9 years old), Entry Level 3 (9–11 years old). After which, there are eight Levels, only two of which are included: Level 1 (GCSE A*–C) and Level 2 (GCSE D–G). This survey does not support an average reading age of 9 as the modal category is Level 2 or above (GCSE A*–C and above), with 56.6% of participants. Only 2.1% of adults surveyed had a reading age of entry level 2, equivalent to 7–9 years of age.

**Table 1:** National qualification framework levels for england and the % of english adults meeting that level in the 2011 skills for life survey.

NQF level	Examples of equivalent qualifications or age	% of the English adult population in the 2011 skills for life survey
8	PhD	Not included
7	Master's, Postgraduate certificate in education (PGCE)	Not included
6	Bachelor's, Graduate diploma	Not included
5	Foundation Degree, Diploma of Higher Education (DipHE)	Not included
4	Certificate of higher education, Higher Apprenticeship	Not included
3	A Level, AS Level, Level 3 national diploma	Not included
2	GCSE A*‐ C, Level 2 national diploma	56.6%
1	GCSE D ‐ G, Level 1 national vocational qualification (NVQ)	28.5%
Entry level 3	Age 9–11	7.8%
Entry level 2	Age 7–9	2.1%
Entry level 1	Age 5–7	5.0%

More recent evidence supports this. The 2023 OECD Survey of Adult Skills uses a different test and scoring system that does not provide an equivalent to reading age [3]. However, it indicates that approximately one in six English adults has low literacy proficiency (Level 1 or below), while the mode and median are at higher proficiency levels. Neither survey supports an average reading age of 9 years.

There is also the issue of ceiling effects when using scoring systems, as shown in the 2011 Skills for Life survey. Some adults would have been capable of scoring higher than a GCSE A*–C grade but are unable to do so due to the scoring limit, which artificially shifts the distribution and makes claims about a mean reading age based on the scores inaccurate.

As the authors cite, there is evidence that health literacy, when considering both literacy and numeracy scores, is a challenge for a large proportion (61%) of the population in one English observational study [4]. However, Rowlands et al. define health literacy as ‘the motivation and ability of individuals to access, understand, and use information in ways which promote and maintain good health’, which is a much broader concept than reading age.

The focus on improving the accessibility of health information is an important one. As the authors identify, the poor readability of regulatory materials risks exacerbating existing health inequalities, as reading ability is unequally distributed as a social determinant of health. However, focusing on reading age shifts the blame onto the individual rather than recognising health literacy as a systemic responsibility. Labelling adults with a child's reading age risks infantilising and stigmatising those who already face health literacy barriers, worsening the very issue we are trying to address. While better metrics are needed, it remains essential to produce documents which are as easily readable as possible, both for ease of use and to make them accessible to all.

As the authors state, readability scores are not a definitive measure; new metrics which include broader elements of comprehension and health literacy would be useful. Current readability scores like Flesch‐Kincaid are linked to US reading ages, which are not directly convertible to UK reading ages. Newer metrics should be validated in large representative samples of the UK population. While these metrics are being developed, a practical approach could be to use the most recent data to describe the percentage of the population likely to be unable to read documents. Haider et al. found that even some of the ‘easy‐read’ documents required reading skills at a tertiary level (NQF level 4) [1]. The Skills for Life report indicates that at least 43.4% of the population would be unlikely to be able to fully understand documents written at a NQF 4 level of complexity. Although the precision of this estimate is limited by using a US reading age conversion, it illustrates the scale of the accessibility challenge.

To address these issues, it is important to have valid metrics and an accurate evidence base. It is concerning that the reading age claim is now being cited as evidence that the average UK reading age is 9 years of age [5]. We believe it is being used by some healthcare professionals as a target and end goal in itself, to the neglect of co‐production and following best practice in the creation of written material. With the increasing availability of artificial intelligence tools, there is a temptation to use these tools to optimise documents to a certain reading age, however, this approach is unvalidated and based on a questionable metric. Co‐production with the intended audience remains crucial, combined with following established guidelines for health information.

## Author Contributions

**George Altman:** conceptualisation (equal), investigation (equal), writing – original draft preparation (lead), writing – review and editing (equal). **Sara Salman:** investigation (equal), writing – review and editing (equal). **Fiona Watson:** conceptualisation (equal), supervision (lead), writing – review and editing (equal). **Mike Oliver:** writing – review and editing (equal). **Gill Rowlands:** writing – review and editing (equal).

## Funding

The authors have nothing to report.

## Conflicts of Interest

The authors declare no conflicts of interest.

## Data Availability

The authors have nothing to report.
